# Effects of two different management systems on hormonal, behavioral, and semen quality in male dromedary camels

**DOI:** 10.1007/s11250-021-02702-6

**Published:** 2021-04-20

**Authors:** Meriem Fatnassi, Barbara Padalino, Davide Monaco, Touhami Khorchani, Giovanni Michele Lacalandra, Mohamed Hammadi

**Affiliations:** 1 Livestock and Wildlife Laboratory, Arid Lands Institute, University of Gabès, 4100 Médenine, Tunisia; 2 Doctoral School of Gabes “SIS”, Rue Omar, Ibn Khattab, 6029 Gabès, Tunisia; 3 Present Address: Department of Agricultural and Food Sciences, University of Bologna, Viale Fanin 44, 40127 Bologna, Italy; 4 Department of Veterinary Medicine, University of Bari Aldo Moro, Bari, Italy

**Keywords:** Hormones, Behaviors, Semen parameters, Female stimulation, Dromedary camel

## Abstract

Effects of two different management systems on male dromedary camel hormones, behaviors, and semen parameters were documented. Camels (*n*=6) were tested under two management systems: (i) housed in single boxes with 1-h freedom (H23); (ii) exposed to females for 17 h (from 3.30 p.m. to 8.30 a.m.) and then housed (ConExF). Blood was collected every morning; camel behavior was recorded twice a day: (i) from 7:00 to 8:00 a.m. to determine the short effects; (ii) from 2:00 to 3:00 p.m. to determine the long effects. Each camel underwent a female parade and semen collection thrice a week; sexual behavior, libido, and semen parameters were assessed. Testosterone and cortisol concentrations were higher in ConExF than H23. Compared to the H23 group, ConExF group spent more time walking, standing tripods, and looking outside their pen/box but they spent less time eating, ruminating, resting, standing, and showing stereotypical behaviors. In the morning, ConExF group spent more time walking, ruminating, and showing typical sexual behaviors compared to themselves during afternoon time and the H23 group. However, in the afternoon time, ConExF camels put more time their heads outside the box through the window and showed higher frequencies of stereotypies, probably due to a higher level of frustration. While the sexual behavioral score was higher and ejaculates showed a higher fraction of milky white and white-colored semen in ConExF than H23 group, their libido was similar. Overall, 17 h of exposure led to an increase in testosterone and cortisol levels, enhancing sexual behavior and semen color, but leading to frustration.

**Supplementary Information:**

The online version contains supplementary material available at 10.1007/s11250-021-02702-6.

## Introduction

The camel population is growing and more intensive camel farms are opening. In intensive farming, camels are often kept in individual boxes with limited opportunities for social contact and other natural behaviors (Fatnassi et al. 2014b; Padalino et al. 2014). However, this management system may lead to sufferance, affecting camel welfare (Padalino et al. 2014; Menchetti et al. 2021). Since welfare is linked with production and reproduction, correct management is crucial for male camels used for artificial insemination. To date, research has focused on the strategies to improve housing conditions of males to enhance their reproductive performance and safeguard their welfare. For instance, increasing space allowance (Hansen and Berthelsen 2000), appropriate diet quality (Thorne et al. 2005), and more opportunity for social contact with conspecifics of the same or different gender (Sondergaard et al. 2011) have been proposed as useful and applicable practices to improve animal welfare. The opportunity for social interaction may be considered an environmental enrichment.

The benefits of social contact of male camels with females on hormones, behavior, and welfare have been reported. In dromedary camels, the daily exposure of male camels to females for 30 min led to an increase in hormonal levels (testosterone and cortisol), frequency of typical rutting behavior, enhancing their welfare status (Padalino et al. 2014). In the literature, the effect of sexual stimulation on hormonal responses has been investigated in males of several species including rams (Rosa et al. 2000) and stallions (Stout 2005). Besides testosterone, it has been reported that cortisol was released after sexual arousal (Colborn et al. 1991) and mating (Villani et al. 2006), suggesting the important role of this hormone in male reproductive function. In our previous study, it was observed that morning cortisol levels decreased in male camels exposed daily to females for 30 min compared to those not exposed at all to females, reflecting the positive effect of social interaction with females on welfare status (Fatnassi et al. 2014b; Padalino et al. 2014). However, the effects of sexual arousal on cortisol are still unclear.

A change in the behavioral repertoire of males has been documented in response to the presence of females. Dromedary camels indeed exhibited more locomotory behavior when housed close to females for 30 min compared to camels housed in single boxes far from females (Bhakat et al. 2005; Fatnassi et al. 2014b), but they showed less appetite and rumination time. The latter authors suggested that the presence of females could stimulate the expression of sexual behaviors because camels became more active and showed all typical rutting behaviors. The effect of sexual arousal on libido and semen parameters was established in stallions (McDonnell 2000) and rams (Fahey et al. 2012). However, these effects were evident only on the first day of stimulation, suggesting that those benefits on ram libido and semen quality were minimal and brief (Fahey et al. 2012). In the new world camelids, Fernandez-Baca (1993) reported an intense sexual activity during the first week of continuous association with females, but this effect was later inhibited despite the presence of estrous females. Up to date, the short-term effects of the female presence on plasma testosterone and cortisol concentrations as well as on the expression of the natural rutting behaviors have been documented (Bhakat et al. 2005; Fatnassi et al. 2014b; Fatnassi et al. 2016). However, to the best of the authors’ knowledge, the effects of prolonged exposure to females on camel libido and semen quality have not been studied yet. Hypothesizing that the exposure to female for 17 h would enhance libido, reproductive performance, and welfare of male camels and those effects would be sustained even in the absence of females, this study aimed at documenting the short- and long-term effects of prolonged exposure to females on males’ testosterone and cortisol levels, behavioral repertoire, libido, and semen characteristics in dromedary camels.

## Materials and methods

### Experimental design

The study was carried out in the peak of the breeding season, starting from January to February 2018, at the Arid Lands Institute’s experimental station in Médenine, Tunisia (33° 30′ N, 10° 40′ E and 18 m above sea level). Six clinically healthy male dromedary camels, ranging in age from 6 to 17 years and with a mean body weight of 545 ± 63 kg, were used. Camels were fed with 6-kg oat hay at 8:30 a.m. and 3-kg concentrate at 1:00 p.m. Animals were watered once every 2 days. The watering and the feeding quantity and quality remained constant throughout the experiment.

The six male dromedary camels were divided into two experimental groups (*n*=3) under a Latin square protocol. Each group was tested under two different management systems: (i) H23 group, each male was housed in a single box (5 ×3 m with 3-m-high solid walls), far away out of sight and smell of the females, for the whole day with 1-h freedom in a paddock (see Fatnassi et al. 2014b) and (ii) ConExF, each male was housed in a separate little pen (150 m^2^) adjacent to the other males and next to the female herd’s pen for 17 h (from 3:30 p.m. to 8:30 a.m.). Each experimental group (*n*=3) was subjected to each management system for 7 days preceded by 2-week habituation period, with a 2-week washout period before being reversed.

The camel herd was composed of females in different physiological states (i.e., receptive, pregnant, and lactating); they were group-housed in a large pen and reared under the semi-extensive system, grazing usually for 7 h from 8:30 a.m. to 3:30 p.m. The males were exposed to females for 17 h when the females’ herd was in their pen.

At 8.30 a.m., when the females went to pasture, ConExF male camels were brought into their single boxes.

All males were filmed twice a day: from 7:00 to 8:00 a.m. and from 2:00 to 3:00 p.m. During the morning period, one group of males was therefore exposed to females and the other was in the box in order to determine the short effect of exposure to females. During the afternoon period, all males were housed in their individual boxes in order to determine the long effect of female exposure.

Behavior was recorded using a video camera system (pro surveillance system, PSS). Cameras were positioned on the female herd’s pen and on each box to provide a complete view. This equipment permitted simultaneous recording of behavior in the two management systems. Immediately after the morning period of video recording, blood samples from both groups (ConExF and H23) were collected daily from the jugular vein into Venoject® tubes with lithium heparin.

Before mating, the sexual behavior of both groups of male camels was scored through a female parade as reported by Fatnassi et al. (2014a). Briefly, the parade lasted a total period of 24 min (4 min/camel). During the parade, male camels were able to touch and sniff the genital area of the female, expressing their sexual behaviors. The male camels previously trained for semen collection using a bovine artificial vagina (30 cm long, 5 cm internal diameter) were subjected to semen collection twice weekly during the habituation period and thrice weekly during the experimental situation. At 9:00 a.m., a receptive female was used to do the parade in front of the males’ boxes as described earlier; then, the technician prepared the female in a couched position in the collection area and the semen collection started. The semen collection session was scheduled as reported in Padalino et al. (2015) (Supplementary Table 1, see Electronic Supplementary Material). The mating behavior of the male camels was directly monitored and scored.

### Hormonal analysis

Within 2 h after sampling, blood was centrifuged at 1000*g* for 15 min at 4 °C and the plasma was stored at −20 °C until analysis. Plasma testosterone concentrations were determined in duplicate by radioimmunoassay (RIA) (Immunotech, Beckman Coulter Company, Ref 1087, Marseille, France). Sensitivity was 0.04 ng/ml and intra- and inter-assay coefficients of variation of the analysis were 7.4% and 11.1%, respectively. Cortisol levels were assessed in duplicate by ELISA method using a commercial cortisol ELISA kit (Demeditec Cortisol ELISA DE 1887, Demetidec Diagnostic GmbH, Kiel, Germany). The absorbance was measured using a Multiscan reader (basic robotic immunoassay operator, BRIO, Radim, Pomezia, Rome, Italy). The intra- and inter-assay coefficients of variation (CV) for plasma cortisol were 5% and 7%, respectively. The results were expressed in ng/ml.

### Behavioral parameters

The videos recorded during the day from 7:00 to 8:00 a.m. and from 2:00 to 3:00 p.m. were analyzed using the ethogram modified from Aubè et al. 2017 (Supplementary Table 2).

The duration of behavioral states (s/60 min) and the frequencies of behavioral events (*n*/60 min) were calculated. The intensities of rutting behaviors (froth on mouth and nervousness) were estimated based on the expression of behaviors such as blathering, dulaa, and teeth grinding.

During the female parade, the occurrences of males’ behavioral events were directly noted down using the ethogram and the sexual behavior (SBS) proposed by Fatnassi et al. (2014a). During the semen collection, direct observation was carried out and the mating behavior was recorded using a focal animal sampling method suggested by Padalino et al. (2015) and applied in Monaco et al. (2015). Briefly, the duration of the behavioral states, the frequency of the behavioral events, the intensity of sexual behavior, and the male’s libido score from 0 (no interest) to 5 (full interest and complete mating time) were recorded.

### Semen parameters

Immediately after semen collection, ejaculates were placed in a water bath at 36 °C and were subjected to the following tests: (1) semen color was evaluated visually and categorized as gray, white, and milky white; (2) volume (ml) was determined directly using a graduated tube; (3) viscosity (cm) was assessed according to the thread test method (Kershaw–Young et al. 2013); (4) mass motility was observed under a phase-contrast microscope (Nikon) at ×100 magnification and was subjectively scored based on the sperm waves through an arbitrary score from 1 (immotile) to 5 (highly motile) (adapted from Monaco et al. 2018); (5) sperm concentration was estimated after dilution of semen with 3% NaCl; the diluted semen was placed into a hemocytometer and the spermatozoa were counted in five squares of one chamber. (6) Viability was evaluated by eosin/nigrosin staining according to Skidmore et al. (2013); 300 spermatozoa were counted, and spermatozoa with unstained heads were counted as viable cells. The total sperm number was calculated as sperm concentration × semen volume.

### Statistical analysis

The normal distribution of data was checked using Kolmogorov-Smirnov test. Testosterone and cortisol concentrations and behavioral repertoire (states and events) were normally distributed and therefore analyzed by GLM. In the model, camels and days were specified as random effects, while management systems (H23 vs. ConExF), time of observations (7:00 a.m. vs. 2:00 p.m.), and their interaction were considered as a fixed effect. Results are expressed as least square means ± standard error (LSM ± SE).

Libido and semen parameters were analyzed by a non-parametric test “Kruskal-Wallis” with the management system as a single factor. Semen color was analyzed by *χ*^2^ test using PROC freq. Results are expressed as mean ± standard error (SE). Statistical analyses were performed using SAS (SAS, v 9.3 2012). The level of statistical significance was set at *P*<0.05.

## Results

### Testosterone and cortisol

The effect of the management systems was significant on testosterone (*df*=1; *F* = 22.81; *P* <0.0001) and cortisol (*df*=1, *F* = 6.92, *P* = 0.009) concentrations. Testosterone and cortisol concentrations were higher in ConExF than in H23 group (T: 16.5 ± 0.9 vs. 12.7 ± 0.9 ng/ml, *P*<0.0001; C: 16.7 ± 0.6 vs. 14.3 ± 0.6 ng/ml, *P* = 0.03, respectively).

### Behavioral repertoire

#### Behavioral states

The interaction between the management system and time of observation was significant only for walking (*df*= 1, *F* = 5.01, *P*=0.02). The effect of management systems (H23 vs. ConExF) was significant on all behavioral states, except for opening legs (*df*=1, *F* = 2.41, *P* = 0.12). Compared to the H23 group, camels in ConExF spent more time walking, standing tripods, and looking outside but less time in eating, ruminating, resting, standing, and showing stereotypy (Table 1).

**Table 1:** Effect of management systems (ConExF vs. H23) on behavioral states recorded during 60 min of observation period in dromedary camels (*n* = 6): housed in a single box for 23 h with 1 h in a paddock (H23), and exposed for 17 h to females (ConExF). Data are expressed as LSM ± SE. Means followed by different letters differ significantly: ^A,B^*P*<0.01; ^a,b^*P*<0.05

Behavioral states (s/60 min)	Management systems		
	H23	ConExF	*P* value
Feeding	801.4 ± 54.0^A^	294.3 ± 54.0^B^	<0.0001
Rumination	209.3 ± 32.5^A^	85 ± 32.5^B^	0.007
Resting	133.6 ± 34.6^a^	8.6 ± 34.6^b^	0.010
Standing	300.7 ± 33.4^A^	107.9 ± 33.4^B^	<0.0001
Walking	4.3 ± 12.7^b^	103.4 ± 12.7^a^	0.030
Stereotypies	889.3 ± 103.4^A^	505 ± 103.4^B^	0.009
Standing opening legs	594.3 ± 72.5	753.6 ± 72.5	0.12
Standing tripods	35.7 ± 30.2^b^	133.6 ± 30.2^a^	0.020
Looking outside	631.4 ± 97.0^B^	1608.6 ± 97.0^A^	<0.0001

**Fig. 1:**
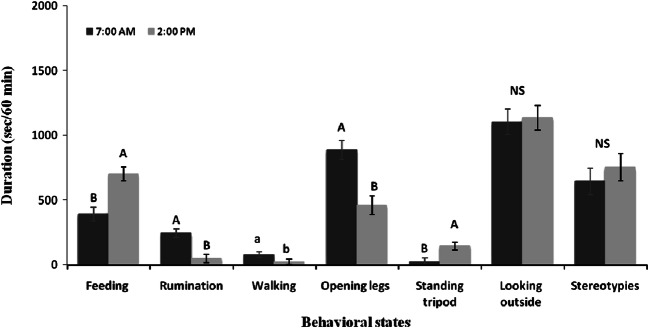
Effect of time of observation (7:00 a.m. vs. 2:00 p.m.) on behavioral states recorded during 60 min of observation period in dromedary camels (*n* = 6). At 7:00 a.m., one group of camels was exposed to the female and the other in the boxes, and at 2:00 p.m., both groups were in the boxes. Data are expressed as LSM ± SEM. Means followed by different letters differ significantly: ^A,B^*P*<0.01, ^a,b^*P*< 0.05

The effect of time of observation (7:00 a.m. vs. 2:00 p.m) was also significant on feeding (*df*=1, *F* = 16.34, *P*<0.0001), rumination (*df*=1, *F* = 17.30, *P*<0.0001), walking (*df*=1, *F* = 5.01, *P*=0.03), opening legs (*df*=1, *F* = 16.34, *P*<0.0001), and standing in tripods (*df*=1, *F* = 8.58, *P*=0.0039). In the morning, camels spent most of their time walking, ruminating, and opening legs compared to the afternoon time. In the afternoon time, camels showed a longer duration of feeding and standing tripods. However, no significant difference was observed between the time of observation (7:00 a.m. vs. 2:00 p.m.) on resting, standing, stereotypy, and looking outside (Fig. 1).

#### Behavioral events

Compared to H23 system, camels in ConExF had a significantly higher frequency of tail flapping (*F* = 11.20, *P*=0.001), flehmen (*F* = 22.36, *P*<0.0001), dulaa (*F* = 28.66, *P*<0.0001), urination (*F* = 5.64, *P*=0.02), interaction with females (*F* = 46.81, *P*<0.0001), and head outside (*F* = 42.19, *P*<0.0001). The intensity of froth on mouth (*F* = 36.45, *P*<0.0001) and nervousness (*F* = 49.76, *P*<0.0001) were also higher in ConExF than in H23. A significant effect (*P*<0.05) of time (7:00 a.m. vs. 2:00 p.m.) was found on the majority of the studied behaviors, except for rolling, opening legs, yawning, scratching, number of steps, and head outside. There was also a significant effect of the interaction management system × time on the frequencies of the following behaviors: urination (*F* = 9.58, *P*=0.002), defecation (*F* = 14.20, *P*=0.0002), tail flapping (*F* = 15.68, *P*=0.0001), flehmen (*F* =21.68, *P*<0.0001), dulaa (*F* = 33.68, *P*<0.0001), number of steps (*F* = 9.49, *P*=0.002), stereotypy (*F* = 5.42, *P*=0.02), interaction with females (*F* = 46.81, *P*<0.0001), and on the intensity of froth on mouth (*F* = 23.89, *P*<0.0001) and nervousness (*F* = 32.36, *P*<0.0001) (Table 2).

**Table 2:** Effect of management system (H23 vs. ConExF), time of observation (7:00 a.m. vs. 2:00 p.m.), and the interaction management system * time on the behavioral events of dromedary camels (*n* = 6): housed in a single box for 23 h with 1 h in a paddock (H23), and exposed for 17 h to females (ConExF). Data are expressed as LSM ± SE

Behavioral parameters		Management system			Time of observation			Interaction
		H23	ConExF	*P* value	7:00 a.m.	2:00 p.m.	*P* value	*P* value
Behavioral frequencies (*n*/60 min)	Head outside	3.5± 0.4	6.9± 0.4	<0.0001	5.4 ± 0.4	0.36	0.36	0.17
	Number of steps	113.6 ± 18.8	162.6 ± 18.8	0.07	158.2 ± 18.8	0.13	0.13	0.002
	Sniffing	2.0 ± 0.3	1.8 ± 0.3	0.72	3.0± 0.3	<0.0001	<0.0001	0.09
	Flehmen	0.1 ± 0.2	1.7 ± 0.2	<0.0001	1.8 ± 0.2	<0.0001	<0.0001	<0.0001
	Defecation	0.9± 0.1	1.01 ± 0.1	0.38	1.7± 0.1	0.007	0.007	0.0002
	Urination	0.9± 0.1	1.3± 0.1	0.02	1.4 ± 0.1	0.0001	0.0001	0.002
	Rubbing/scratching	2.4 ± 0.3	2.6 ± 0.3	0.62	2.8 ± 0.3	0.17	0.17	0.06
	Scratching occipital glands	0.8± 0.1	1.1 ± 0.1	0.10	1.2 ± 0.1	0.006	0.006	0.53
	Yawning	1.4 ± 0.3	1.7 ± 0.3	0.52	1.5 ± 0.3	0.87	0.87	0.26
	Dulaa	0.9 ± 0.5	4.9± 0.5	<0.0001	4.3 ± 0.5	0.0002	0.0002	<0.0001
	Tailflapping	7.7 ± 6.7	39.6 ± 6.7	0.001	36.3 ± 6.7	0.009	0.009	0.0001
	Open leg	3.1± 0.4	3.6± 0.4	0.30	3.6 ± 0.4	0.19	0.19	0.89
	Rolling	0.2 ± 0.1	0.1± 0.1	0.06	0.1 ± 0.1	0.52	0.52	0.11
	Stereotypies	39.7 ± 9.2	36.5± 9.2	0.80	24.2 ± 9.2	0.03	0.03	0.020
	Interaction with male camels	1.1± 0.3	1.6 ± 0.3	0.15	1.8 ± 0.3	0.008	0.008	0.92
	Interaction with females	0.01 ± 0.4	3.5 ± 0.4	<0.0001	3.5 ± 0.4	<0.0001	<0.0001	<0.0001
Behavioral intensities (score 1 to 5)	Froth on the mouth	1.0 ± 0.1	1.5± 0.1	<0.0001	1.4 ± 0.1	<0.0001	<0.0001	<0.0001
	Nervousness	1.5 ± 0.1	2.6 ± 0.1	<0.0001	2.3± 0.1	0.03	0.03	<0.0001

The results of the behavioral events recorded during the morning (from 7:00 to 8:00 a.m.) and afternoon observations (from 2:00 to 3:00 p.m.) are shown in Supplementary Material (Supplementary Table 3). It is worth noting that the frequency of the typical sexual behavior (e.g., flehmen, defecation, urination, dulaa extrusion, tail flapping) recorded during morning observation (from 7:00 to 8:00 a.m.) was higher in ConExF group compared to H23 group. The intensities of frothing on mouth and nervousness were also higher (*P*< 0.0001). However, compared to ConExF group, the frequencies of stereotypies and rolling were significantly higher in the H23 group. During the afternoon period (from 2:00 to 3:00 p.m.) and while all camels were in their single boxes, the frequencies of head out, stereotypies, and interaction with male camels were significantly higher in ConExF group compared to H23 group. However, the frequency of sniffing and defecation was significantly higher in H23 than in the ConExF system.

### Sexual behavior score

**Fig. 2:**
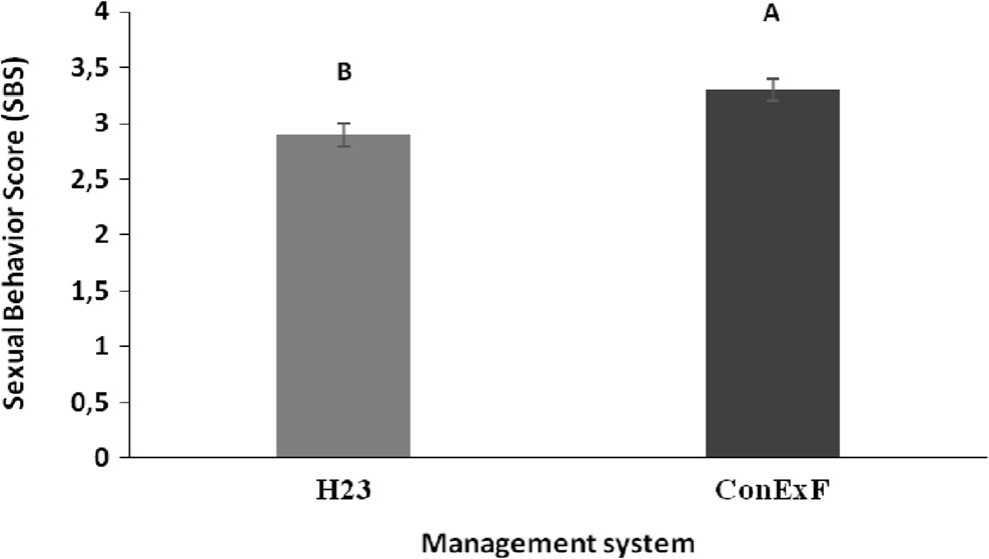
Sexual behavior score (SBS: 1–5) of dromedary camels (*n* = 6) housed in two different management systems (H23 vs. ConExF): housed in a single box for 23 h with 1 h in a paddock (H23), and exposed for 17 h to females (ConExF). Data are expressed as LSM ± SEM. Means followed by different letters differ significantly: ^A, B^*P*< 0.01

Sexual behavior score (SBS) of housed male’s dromedary camels was significantly affected (*df*=1; *F* = 8.85; *P*=0.0043) by the management systems (Fig. 2). It was higher in males housed in the ConExF system (3.3 ± 0.1) compared to H23 (2.9 ± 0.1) group.

### Libido and semen quality

**Fig. 3:**
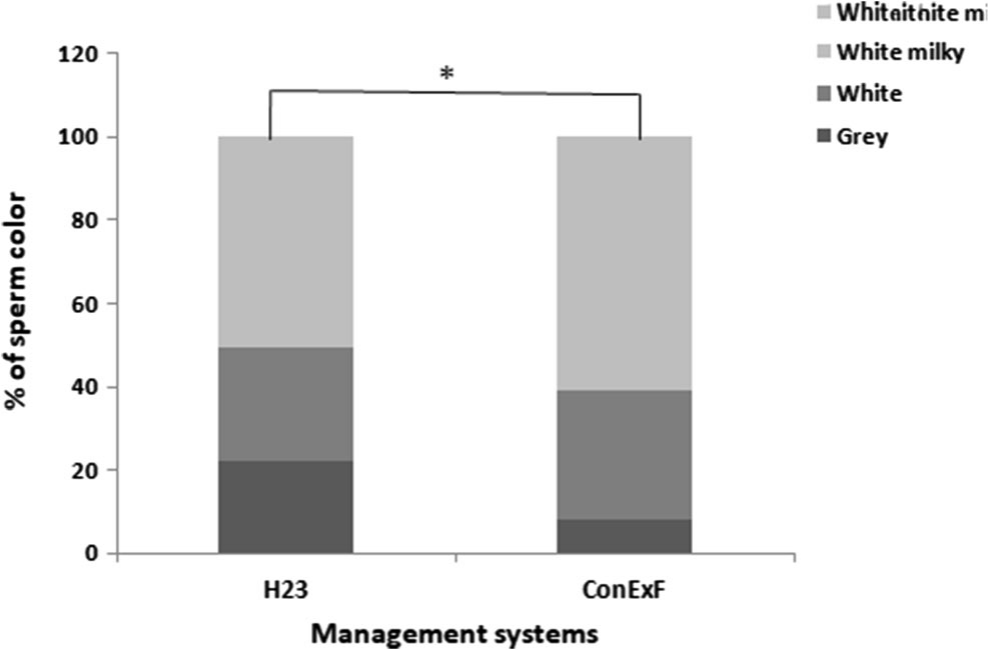
Variation in the relative frequency of semen color in two different management systems (H23 vs. ConExF): housed in a single box for 23 h with 1 h in a paddock (H23), and exposed for 17 h to females (ConExF). *P<0.05

There was no difference in the libido and mating behavior between ConExFand H23 camels (*P*>0.05) (Table 3). The color of semen varied instead significantly (*P* =0.013) between the two management systems (Fig. 3). Semen volume was higher in male camels housed in H23 compared to ConExF (*P* < 0.05), but sperm concentration tended to increase (*P* = 0.06) in ConExF camels. No other differences were observed (Table 4).

**Table 3:** Duration and frequencies of recorded parameters during semen collection session of housed dromedary camels (*n* = 6) in two different management systems (H23 vs. ConExF): housed in a single box for 23 h with 1 h in a paddock (H23), and exposed for 17 h to females (ConExF). Data are expressed as mean ± SE

Parameters	H23	ConExF	*P* value
Latency time (s; LT)	175.9 ± 81.4	33.9 ± 6.5	0.06
Service time (min; ST)	13.3 ± 2.8	12.3 ± 2.5	0.75
Time spent near female/standing over the female (min; TSNF)	10.2 ± 2.1	12.4 ± 2.0	0.44
Time spent far to female/walking around (min; TSFF)	12.5 ± 2.6	17.3 ± 2.1	0.11
Mating time (min; MT)	36.1 ± 4.1	42.1 ± 1.7	0.17
Blatering (*n*/TSNF)	19.9 ± 5.0	18.5 ± 5.1	0.82
Teethgrinding (*n*/TSNF)	80.5 ± 37.6	74.2 ± 32.8	0.87
Tailflapping (*n*/TSNF)	2.3 ± 0.9	6.6 ± 4.4	0.34
Sound emission (*n*/TSNF)	5.5 ± 2.5	3.7 ± 1.8	0.57
Sniffing (*n*/TSNF)	4.2 ± 1.4	4.4 ± 1.1	0.88
Flehmen (*n*/TSNF)	0.5 ± 0.2	0.2 ± 0.1	0.33
Defecation (*n*/TSNF)	0.7 ± 0.3	0.8 ± 0.5	0.81
Dulaa (*n*/TSNF)	19.4 ± 3.6	13.7 ± 2.6	0.06
Yawning (*n*/TSNF)	0.4 ± 0.2	0.6 ± 0.4	0.64
Contact/interaction with female (*n*/TSNF)	3.9 ± 1.7	3.5 ± 0.9	0.87
Number of mounts (*n*/MT)	4.3 ± 0.8	4.5 ± 0.7	0.84
Attempt of mounts (*n*/MT)	1.1 ± 0.3	1.3 ± 0.5	0.64
Froth on the mouth (score 1 to 5)	2.9 ± 0.4	2.9 ± 0.4	0.81
Nervousness (score 1 to 5)	2.7 ± 0.3	2.8 ± 0.2	0.64
Libido score (0 to 5)	3.0 ± 0.4	3.5 ± 0.2	0.21

**Table 4:** Effect of two different management systems (H23 vs. ConExF) on semen quality of housed dromedary camels (*n* = 6): housed in a single box for 23 h with 1 h in a paddock (H23), and exposed for 17 h to females (ConExF). Data are expressed as mean ± SE

Semen parameters	H23	ConExF	*P* value
Volume (ml)	13.5 ± 1.9	11.5 ± 2.0	0.020
Viscosity (cm)	6.7 ± 0.9	7.0 ± 0.7	0.28
Mass motility (0 to 5)	2.4 ± 0.3	2.6 ± 0.3	0.75
Viability (%)	52.6 ± 4.0	52.0 ± 3.5	0.84
Concentration (×10^6^SPZ/ml)	293.1 ± 67.5	414.5 ± 65.0	0.06
Total number of SPZ (×10^6^)	3956.8 ± 128.3.9	4766.7 ± 130.7	0.17
Total number of viable SPZ (×10^6^)	2081.3 ± 378.0	2478.7 ± 207.1.	0.21

## Discussion

This study documented the effects of 17 h of exposure to females on testosterone and cortisol levels, behavioral repertoire, libido, and semen quality in male dromedary camels. The data supported partially our hypothesis. We found an increase in testosterone and cortisol levels recorded at 8:00 a.m. in the ConExF group compared to the H23 group. The exposure of male dromedary camels to the females had a clear short-term effect on their behavior, as expected. Male camels exposed to the females in the morning behaved in a completely different way compared to the camels housed individually. However, we failed to demonstrate that the exposure for 17 h to a female herd would have a positive long effect (i.e., in absence of the female), enhancing the libido and semen quality. Our results have increased our knowledge of the complex relationship between housing systems, welfare status, and reproductive performance in male camels.

ConExF camels had a higher level of testosterone and cortisol at 8:00 a.m. after being exposed for 17 h to females. The testosterone response is in line with the literature (Rosa et al. 2000; Christensen et al. 2009). A similar rise of testosterone secretion was previously described in dromedary camels (Bhakat et al. 2005). Males respond to female stimulation with an increase in luteinizing hormone (LH) immediately followed by a rise in plasma testosterone (Stout 2005; Villani et al. 2006). However, it has also shown that the presence of a receptive female was not always necessary to increase testosterone level, because the presence of a female in any physiological stage was sufficient to elicit a similar testosterone rise (Johnston and Bronson 1982). The increase in cortisol level was similar to previous results found in boars (Borg et al. 1991), stallions (Colborn et al. 1991; Villani et al. 2006), and dromedary camels (Bhakat et al. 2005). Findings suggested that cortisol is implicated in sexual stimulation in males, but the effects of sexual excitation on cortisol level are still conflicting. Indeed, it has been suggested that sexual arousal might lead to an increase in cortisol useful for accelerating the protein and carbohydrate metabolism and mobilizing energy toward the pursuit of sexual activity (Veronesi et al. 2011). However, other studies reported that sexual stimulation may decrease psychological stress, leading to a decline in circulating cortisol levels in dromedary camels (Fatnassi et al. 2014b) and rodents (Devries et al. 2007). The latter authors suggested that the reduced cortisol response might reflect the ameliorative status of males due to the closer presence of conspecifics, supposing an antagonistic relationship between cortisol and sexual arousal. In our case, the increase in cortisol may also be related to the increase of the locomotory behavior shown by the ConExF male camels. The relationship between cortisol levels, housing system, and sexual arousal in camels warrants further investigations.

There was no effect of the interaction between the management system*time on the majority of behavioral states. This could be due to the absence of difference in camels’ behavior during the afternoon observations (2:00 p.m.) since they all were inside the boxes or for the small sample size we used. However, the fixed effects of management and time were significant and expected. It was indeed not surprising that direct exposure to females induced a change in male camels’ behavioral repertoire. In the morning, while exposed to the females, ConExF male camels spent more time walking, standing tripods, and looking outside, and less time eating, standing, ruminating, and showing stereotypical behaviors compared to H23 group. Those findings are in line with previous studies (Bhakat et al. 2005; Fatnassi et al. 2014b; Fatnassi et al. 2016). When reared in a larger space and stimulated by the presence of female camels, our males were probably able to meet their need of social and locomotory behaviors, as previously suggested (Fatnassi et al. 2014b; Padalino et al. 2014). However, ConExF camels ate less and showed a prolonged tripod position and increased aggression toward the other males. The limited-time spent in feeding and ruminating could be partly due to the feeding practices; in our experimental center, camels were fed twice a day at fixed times with a predetermined quantity of oat hay (6 kg at 8:30 a.m.) and concentrate (3 kg at 1:00 p.m.). However, it may also be considered typical behavior of male camels in rut; indeed, Bhakat et al. (2005) noticed that rutting camels reduced eating and lost their body weight (approximately 25%) by the end of the breeding season. Standing in a tripod position might be a sign of frustration due to the fact that they were close to females but unable to reach them for mating because of the wall barrier. Increased aggressivity was expected since it has been already reported that social contact with the same-sex provokes an increase in social interaction and aggression in camels grouped in high stocking density (EL Shoukary et al. 2020).

The frequencies and the intensities of sexual behavioral recorded during the morning observation were higher in ConExF male camels than in the H23 group. This is in agreement with the literature (Bhakat et al. 2005; Fatnassi et al. 2014b), reporting that the presence of females stimulates the expression of typical sexual behavior in rutting camels. In agreement with studies in other species, it seems that the sexual stimulation through 17-h exposure to females (ConExF) had a short-term effect on the sexual behaviors of the camels, as shown by the findings related to the sexual behavior score registered during the female camel parade. However, contrary to our expectations, no significant differences were found in the frequencies of typical sexual behavior in both groups during the afternoon period. Moreover, our results showed a higher frequency of head out behavior, stereotypies, and interaction with male camels in ConExF than H23 camels. The expression of these behaviors was probably related to the absence of females, suggesting that when rehoused in their single boxes, camels felt frustrated. Therefore, it appears that the effect of 17 h of female’s exposure on behavioral repertoire was short and counterproductive since camels showed behavior related to stress (i.e., stereotypic behaviors) after re-housing in their single boxes. Our findings are in disagreement with studies conducted in boars, reporting a positive and permanent effect of continuous exposure to females on their sexual behaviors (Hemsworth et al. 1981; Hemsworth and Tilbrook 2007).

Our study showed that 17 h of exposure to females had also minimal benefits in terms of camel’s libido. The lack of significant improvement of male’s libido may be explained by the period of this experiment; indeed, our study was conducted during the peak of the rutting season in Tunisia (January–February), in which all tested male camels were sexually active having a good libido. Moreover, before semen collection, both groups were stimulated using a parade of a receptive female, which could have been a good stimulus to enhance the libido in the H23 group. Apart from an enhancing of the semen color and a trend of increasing in sperm concentration in ConExF group, semen parameters of both groups were also similar, except for semen volume which was higher in H23 group. However, all parameters were within normal range and in line with the literature (Allam et al. 2013; Monaco et al. 2018). In general, semen color of dromedary camels varied from gray to milky white or creamy and this variation may be due to the different concentrations of spermatozoa and semen consistency (Allam et al. 2013). The abundance of the milky white color in semen collected from camels housed in ConExFgroup could be associated with sperm concentration (Fatnassi 2017). Our results were partly in line with Fahey et al. (2012), reporting a significant increase in sperm concentration in exposed rams to females. However, the lack of other differences did not support our hypothesis and was unexpected. This might be due to the low number of camels used or to the high frequency of semen collections we carried out in this study. However, this hypothesis needs to be further investigated comparing the effect of semen frequency (twice vs. three times weekly) on semen parameters of male camels.

Notwithstanding the before-mentioned limitations, our findings have increased our understanding of dromedary camel reproductive physiology and behavior, and might be useful to propose more efficient management to enhance the welfare of male camels used for artificial insemination.

Overall, this study documented that 17 h of exposure to females led to an increase in testosterone and cortisol levels, modification of behavioral repertoire, enhancing sexual behavior and semen color. However, considering that camels did not eat when exposed to the females, got frustrated when rehoused, and showed similar libido and semen quality, it seems that stimulating males with females for so many hours was not favorable. Giving them 1-h freedom in the paddock and regular short contact with females seemed to be beneficial for male camel welfare and libido.

## Supplementary Information

ESM 1 (DOCX 24 kb)

## Data Availability

On request
