# Supplementary material for: Effects of two different management systems on hormonal, behavioral, and semen quality in male dromedary camels
Source: Trop Anim Health Prod. 2021 Apr 20;53(2):275. doi: 10.1007/s11250-021-02702-6 (PMC8057974; doi:10.1007/s11250-021-02702-6)
Supplement: Supplementary file 1 — (DOCX 24 kb) [file 11250_2021_2702_MOESM1_ESM.docx]

**The effect of two different management systems on hormonal, behavioral and semen quality in male dromedary camels**

**Tropical Animal Health and Production**

Meriem Fatnassi^1,4^, Barbara Padalino^2a^, David Monaco^3^, Touhami Khorchani^1^, Giovanni Michele Lacalandra^3^ and Mohamed Hammadi^1,4^

^1^Livestock and Wildlife Laboratory, Arid Lands Institute, 4100 Médenine, University of Gabès, Tunisia

^2^Department of Agricultural and Food Sciences, University of Bologna, VialeFanin 44, 40127, Bologna, Italy

^3^Department of Veterinary Medicine, University of Bari Aldo Moro, Italy

^4^Doctoral School of Gabes “SIS”, Rue Omar Ibn Khattab 6029 Gabès

^a^***Present address:***

Corresponding author: Barbara Padalino. E-mail: barbara.padalino@unibo.it

**Table 1** Maximal timing used during semen collection procedure of dromedary camel bulls

| **Parameters** | **Description** | **Maximal time (min)** |
| --- | --- | --- |
| **Latency time** | Time from the exits of camel from the box until its mount for the first time. | 15 |
| **Time between two services** | Time from withdrawal of penis from artificial vagina until its intromission again. | 20 |
| **Standing on/over the Female time** | Time spent when the male camel is near female expressing their sexual behaviors (sniffing, flehmen, dulaa…). | 30 |
| **Walking around time** | Time when the camel is walking in the collection area, being not interested in, and far from, the female. | 4 × 3 |
| **Mating time** | The time from first sitting on female to the return in the box= service/ejaculation time + standing over the female + walking around. | 45 |

**Table 2** Ethogram used to score the video for behavioral sates and events (modified from Aubé et al. 2017)

| Behavior | Description |  |
| --- | --- | --- |
| *Behavioral states*(s/60 min) | | |
| Feeding | Camel takes food into his mouth (hay or concentrate), chews and swallows it. |  |
| Rumination | A bolus goes back into his mouth and the camel chews it. |  |
| Walking | Camel does more than 2 complete steps. |  |
| Standing | Camel stands on his four feet in the box or apparentlydoes nothing. |  |
| Standing in tripods | Camel stands on his three feet and looks outside the box |  |
| Looking outside | Camels looks with head outsiders from the bars of the box or the wall |  |
| Opening legs | Camel stands and opens his posterior legs with an angle > 45°C |  |
| Lying down | Camel sits in sternal recumbency: sitting up on the brisket with the legs tucked under the body, the natural sitting position in camel, with head on the floor or not. Camel rests or sleeps and does nothing else. |  |
| Stereotypy | **Wall-licking**: Repetitive licking of the wall (tongue movements with contact on the wall of the box) |  |
|  | **Box walking**: Walking around and round in the box in the same direction, in alternate directions or in a figure of 8. Behavior is repeated regularly and in an unvaried manner. |  |
|  | **Swaying**: Camel sways his body slightly from one side to the other (i.e. Camel remains stationary but shifts his weight from one foreleg to the other and swings his head from side to side).  **Self-mutilation:** The camel bitted his own forelegs (right or left) at different part of the legs (to the shoulder to the feet)" |  |
| *Behavioral events*(n/60 min) | | |
| Head out | Camel puts his whole head, or part of it, through a window or through the bars on the door. |  |
| Sniffing | Exploration of the environment bringing the nose into contact with an object (e.g. the wall of the box). |  |
| Sound emission | Camel emits a sound from his mouth (sound is different from blathering). |  |
| Defecation | Elimination of feces. |  |
| Urination | Elimination of urine. |  |
| Scratching | Camel scratches a part of his body on external object (bars, wall, floor…) or with another body part (e.g. scratches his head with his foot). |  |
| Yawning | Involuntary sequence consisting of mouth opening, deep inspiration, brief apnea, and slow expiration. |  |
| Open leg | Camel splays his hind legs until they form an angle of at least 45°. |  |
| Interaction with male | Camel comes into physical contact with another neighboring male (usually nose-to-nose contact, over the wall separating two boxes or through a window). |  |
| Interaction with female | Camel comes int o physical contact with females; usually nose, smell and touch females without full contact (mating) |  |
| Stereotypy | **Bar mouthing**: Licking, biting or playing with the lips and/or tongue on the bars of the door. |  |
|  | **Head shaking**: Camel bends his necks backwards in a very fast movement (including head movements of up to 90°). This stereotypy was considered as a behavioral event because it lasted only about one second. |  |
| Sexual behaviors | **Scratching occipital gland**: Camel scratches his occipital gland on any surface (floor, wall …) in order to spread the dark secretion of the occipital poll glands. |  |
|  | **Teeth grinding**: Camel moves his lower jaw left and right, with mouth closed, grinding the teeth and producing a typical squeaking/whistling sound. |  |
|  | **Blathering**: Emission of typical gurgling and roaring sounds. |  |
|  | **Dulaa extrusion**: Exteriorization of the soft palate, usually known as the “dulaa”. |  |
|  | **Flehmen**: Camel lifts his head and curls his upper lip. |  |
|  | **Tail flapping**: The tail is held under the prepuce opening for a few seconds, and then, it is beaten up and down several times, usually spreading urine over the croup and surrounding areas. |  |
| *Behavioral intensities* (score from 1 (absent) to 5 (very high)) | | |
| Froth on mouth | Presence of a foam of saliva in in mouth |  |
| Nervousness | Increased pacing, anxiety and sound loading |  |

**Table 3** Effect of 17 hours of exposure to females’ herd on behavioral frequencies and intensities of dromedary camels (n = 6) compared to housing in H23 management system recorded during 60 min of the morning observation (from 7:00 to 8:00 a.m) and in the afternoon period (from 2:00 to 3:00 p.m); while all camels were in their single boxes. Data are expressed as LSM± SEM. Mean followed by different letters differ significantly: ^A,B^: *P<0.01*,^a,b^: P< 0.05.

|  | | ***Management system*** | | | | | |
| --- | --- | --- | --- | --- | --- | --- | --- |
|  |  | **7:00 to 8:00 a.m** | | | **2:00 to 3:00 p.m** | | |
|  |  | **H23** | **ConExF** | **P value** | **H23** | **ConExF** | **P value** |
| **Behavioral frequencies**  **(n/60 min)** | Head outside | 3.4 ± 0.5^B^ | 7.5 ± 0.5^A^ | <0.0001 | 3.6 ± 0.5^B^ | 6.3 ± 0.5^A^ | 0.0001 |
|  | Number of steps | 92.9 ± 26.5^B^ | 223.6 ± 26.5^A^ | 0.003 | 134.4 ± 12.5 | 101.7 ± 12.5 | 0.07 |
|  | Sniffing | 2.7 ± 0.4 | 3.2 ± 0.4 | 0.43 | 1.3 ± 0.2^A^ | 0.5 ± 0.2^B^ | 0.009 |
|  | Flehmen | 0.3 ± 0.3^B^ | 3.4 ± 0.3^A^ | <0.0001 | 1.0 ± 0.1^A^ | 0.5 ± 0.1^B^ | 0.006 |
|  | Defecation | 0.8 ± 0.2^B^ | 1.6 ± 0.2^A^ | 0.007 | 0.8 ± 0.1 | 0.7 ± 0.1 | 0.52 |
|  | Urination | 0.9 ± 0.2^B^ | 1.9 ± 0.2^A^ | 0.0006 | 1.8 ± 0.4 | 2.7 ± 0.4 | 0.08 |
|  | Rubbing/Scratching | 3.1 ± 0.4 | 2.5 ± 0.4 | 0.26 | 0.5 ± 0.2 | 0.8 ± 0.2 | 0.36 |
|  | Scratching occipital glands | 1.0 ± 0.2 | 1.5 ± 0.2 | 0.16 | 1.2 ± 0.5 | 2.1 ± 0.5 | 0.15 |
|  | Yawning | 1.7 ± 0.3 | 1.4 ± 0.3 | 0.63 | 1.6 ± 0.3 | 1.3 ± 0.3 | 0.41 |
|  | Dulaa | 0.2 ± 0.7^B^ | 8.5 ± 0.7^A^ | <0.0001 | 0.1 ± 0.1 | 0.1 ± 0.1 | 0.66 |
|  | Tailflapping | 1.4 ± 9.5^B^ | 71.2 ± 9.5^A^ | 0.0001 | 14.0 ± 2.8 | 8.1 ± 2.8 | 0.15 |
|  | Open leg | 3.4 ± 0.5 | 4.0 ± 0.5 | 0.23 | 2.8 ± 0.5 | 3.2 ± 0.5 | 0.57 |
|  | Rolling | 0.3 ± 0.1^a^ | 0.1 ± 0.1^b^ | 0.02 | 0.2 ± 0.1 | 0.2 ± 0.1 | 0.81 |
|  | Stereotypies | 41.0 ± 4.2^A^ | 7.4 ± 4.2^B^ | <0.0001 | 38.4 ± 8.4^b^ | 65.5 ± 8.4^a^ | 0.02 |
|  | Interaction with male camels | 1.6 ± 0.4 | 2.1 ± 0.4 | 0.44 | 0.5 ± 0.2^b^ | 1.1 ± 0.2^a^ | 0.04 |
|  | Interaction with females | 0.1 ± 0.5^B^ | 7.1 ± 0.5^A^ | <0.0001 | 0.1 ± 0.1 | 0.1 ± 0.1 | 0.65 |
| ***Behavioral intensities (*score 1 to 5)** | Froth on the mouth | 1.0 ± 0.1^B^ | 1.9 ± 0.1^A^ | <0.0001 | 1.0 ± 0.1 | 1.1 ± 0.1 | 0.19 |
|  | Nervousness | 1.3 ± 0.2^B^ | 3.3 ± 0.2^A^ | <0.0001 | 1.8 ± 0.2 | 2.0 ± 0.2 | 0.30 |
